# Remodeling of the *Streptococcus mutans* proteome in response to LrgAB and external stresses

**DOI:** 10.1038/s41598-017-14324-w

**Published:** 2017-10-25

**Authors:** Sang-Joon Ahn, Tongjun Gu, Jin Koh, Kelly C. Rice

**Affiliations:** 1 Department of Oral Biology, College of Dentistry, University of Florida, Gainesville, FL 32610 USA; 2 Bioinformatics, Interdisciplinary Center for Biotechnology Research, University of Florida, Gainesville, FL32610 USA; 3 Proteomics and Mass Spectrometry, Interdisciplinary Center for Biotechnology Research, University of Florida, Gainesville, FL32610 USA; 4 Department of Microbiology and Cell Science, Institute of Food and Agricultural Sciences, University of Florida, Gainesville, FL 32611 USA

## Abstract

The *Streptococcus mutans* Cid/Lrg system represents an ideal model to study how this organism withstands various stressors encountered in the oral cavity. Mutation of *lrgAB* renders *S*. *mutans* more sensitive to oxidative, heat, and vancomycin stresses. Here, we have performed a comprehensive proteomics experiment using label-free quantitative mass spectrometry to compare the proteome changes of wild type UA159 and *lrgAB* mutant strains in response to these same stresses. Importantly, many of identified proteins showed either a strikingly large fold-change, or were completely suppressed or newly induced in response to a particular stress condition. Notable stress proteome changes occurred in a variety of functional categories, including amino acid biosynthesis, energy metabolism, protein synthesis, transport/binding, and transcriptional/response regulators. In the non-stressed growth condition, mutation of *lrgAB* significantly altered the abundance of 76 proteins (a fold change >1.4, or <0.6, *p*-value <0.05) and several of these matched the stress proteome of the wild type strain. Interestingly, the statistical correlation between the proteome changes and corresponding RNA-seq transcriptomic studies was relatively low (*rho*(ρ) <0.16), suggesting that adaptation to a new environment may require radical proteome turnover or metabolic remodeling. Collectively, this study reinforces the importance of LrgAB to the *S*. *mutans* stress response.

## Introduction

The pathogenic potential of *Streptococcus mutans*, a major etiological agent of human dental caries^1^, is intimately linked to its ability to efficiently and rapidly adjust to the adverse environment of the oral cavity, thus improving its survival and persistence in dental plaque biofilm^2,3^. In this regard, considerable work has focused on the *S*. *mutans* Cid/Lrg system, consisting of two dicistronic operons *lrgAB* (SMU.575c/574c) and *cidAB* (SMU.1701c/1700c)^4–7^. Expression of *cid* and *lrg* is highly responsive to complex and unfavorable environmental signals that integrate into the regulatory networks modulating *S*. *mutans* virulence. For example, *lrg* and *cid* genes display opposite patterns of expression in response to growth phase, glucose concentration, oxygenation, and blood plasma^6,8^ and are transcriptionally cross-regulated^5,6^. Regulation of the *lrg* and *cid* operons is further complicated by involvement of multiple major regulators, including CcpA (carbon catabolite protein A) and the TCSs (two-component signal transduction systems) LytST and VicKR^4–6^. Consequently, the Cid/Lrg system affects comprehensive virulence traits such as autolysis, biofilm development, oxidative and heat stress response, antibiotic resistance and genetic competence^4–6^ required for successful colonization in the oral cavity. For these reasons, the Cid/Lrg system has potential as an attractive target for development of anti-caries treatments.

Another value in studying the Cid/Lrg system is that *lrgA* and *cidA* encode membrane proteins with predicted similarity to bacteriophage holin: antiholin proteins that modulate cell lysis. Holins are known to control the timing and onset of host cell lysis during bacteriophage lytic infection, and thus it has been hypothesized that Cid and Lrg may contribute to inducing death and/or lysis in a subpopulation of the bacterial community for the altruistic benefit of the entire population, as a survival strategy for adapting to environmental conditions^9–11^. Conclusive data proving that the Cid/Lrg systems of *S*. *mutans* and *S*. *aureus* are functionally analogous to holins/antiholin pairs is still lacking. However, published *S*. *mutans* data strongly suggest that Cid and Lrg proteins are functionally interrelated^5,7^. For example, we recently found that the lack of either CidB or LrgAB results in very similar phenotypes with respect to ultrasensitivity to aeration, heat, and vancomycin stress^5,7^. More interestingly, our recent RNA-seq data has also demonstrated almost identical transcriptome changes between *cidB* and *lrgAB* mutants, compared to wild type, underlining likely functional and mechanistic similarities between CidB and LrgAB^5,7^. However, molecular mechanisms for how these encoded proteins modulate *S*. *mutans* virulence and its stress response are still not well understood. As well, clues to their specific cellular functionality are relatively scarce due in part to the inherent difficulties associated with studying membrane proteins.

“-Omics” technologies have provided a powerful means of broadly and globally assessing the cellular responses and adaptation of bacteria to stresses. We recently performed a comprehensive RNA-seq experiment to assess the transcriptomic changes of wild-type and isogenic *lrgAB* mutants under anaerobic (control) and stress-inducing culture conditions (aerobic, heat and vancomycin stress), as a way to connect known stress-sensitive phenotypic aspects to information regarding specific changes in gene expression^7^. These environmental stresses and *lrgAB* mutation both influenced the transcriptome in *S*. *mutans*, with implications for bacterial cell death/lysis, adaptation and virulence. Nevertheless, understanding transcriptomic changes alone may be insufficient for defining the exact dynamic cellular changes of *S*. *mutans* in response to environmental stresses.

This present study investigates the changes in proteome profiles that occurred in the same set of *S*. *mutans* wild-type and *lrgAB* mutant samples used in our recently published RNA-seq study^7^. To this end, mass spectrometry-based label-free quantitative proteomics^12–15^, a technology known to enable comprehensive identification and quantification of complete bacterial proteomes, was adapted to study changes in *S*. *mutans* intracellular protein levels in response to aeration, heat, and vancomycin stress. Furthermore, the degree of correlation between *S*. *muta*ns protein abundance profiles and gene expression changes was determined. Further understanding of these combined “-omics” data at the cellular or molecular level will enhance our knowledge of Cid/Lrg-mediated cellular responses of *S*. *mutans* to adverse environments. Furthermore, these “-omics” data will serve as a valuable resource that can be mined to help clarify the role of the *S*. *mutans* stress response and physiological activity to its dynamic survival in the oral cavity.

## Materials and Methods

### Bacterial strains and media

The bacterial strains used in this study were *S*. *mutans* UA159, a serotype c strain^16^, and a previously published isogenic *lrgAB* mutant^6^, which was created using the PCR ligation mutagenesis technique^17^. Both *S*. *mutans* strains were grown and maintained on Brain Heart Infusion (BHI) agar plates (containing 1,000 µg/ml kanamycin for the *lrgAB* mutant), at 37 °C and 5% CO_2_.

### Bacterial cultures for protein preparation

The identical set of bacterial cultures analyzed in our recently-published RNA-seq work^7^ were also used in this present study. Briefly, wild type strain UA159 (designated “WT” in all subsequent figures and tables) and Δ*lrgAB* (designated “AB” in all subsequent figures and tables) overnight cultures were each diluted 1:50 in 50 ml sterile BHI broth and grown to mid-exponential growth phase (OD_600_ = 0.4) under four different environmental conditions (anaerobic, (control); aerobic, (a); heat stress, (h); and vancomycin, (v) stress), as follows: For anaerobic growth, including heat and vancomycin stress conditions, sterile mineral oil was placed on top of the cultures. For aerobic growth, each culture was grown in a 250-ml conical flask at a 1:5 volume to flask ratio, and incubated at 115 rpm and 37 °C. For heat stress growth, cultures were incubated at 40 °C. For vancomycin growth, cultures were supplemented with 1 µg/ml vancomycin and incubated at 37 °C. An aliquot (5 ml) of each culture was previously used for the RNA-seq experiment^7^, and the rest (45 ml) of the culture was subjected to the mass spectrometry experiments in this study. Growth curves of these strains under each condition can be found in our previous publication^7^.

### Protein Extraction and Quantification

Biological pentaplicates (i.e. *n* = 5 replicates per *S*. *mutans* strain per growth condition, including the n = 3 replicates analyzed previously by RNA-seq.^7^) of each *S*. *mutans* culture were processed for protein extraction according to Fujiki *et al*.^18^ with the following modifications: Samples were ground into fine powder in liquid nitrogen and agitated in extraction buffer (0.1 M Tris-HCl pH 8.8, 10 mM EDTA, 0.2 M DTT, 0.9 M sucrose) with an equal volume of phenol (pH 8.0) for 2 hours at room temperature. The phenol phase was precipitated by adding five volumes of 0.1 M ammonium acetate in methanol. After washing twice with 0.1 M ammonium acetate in methanol and twice with 80% acetone, the dried pellet was dissolved with 50 mM ammonium bicarbonate buffer. The mixtures were incubated on ice before centrifugation at 4 °C for 20 minutes at 12,000 × g, separating the proteins into soluble and insoluble phases. The insoluble phase was incubated with protein buffer (8 M urea, 2 M thiourea, 2% ASB-14 (amidosulfobetaine 14)) for at least one hour with occasional vortexing. The proteins were quantified using an EZQ Protein Quantitation Kit (Invitrogen, Carlsbad, CA, USA) and SoftMax Pro Software v5.3 (Molecular Devices, Downingtown, PA, USA).

### Protein Digestion and LC-MS/MS

Proteins were precipitated in 25 mM ammonium bicarbonate, pH 8.0 with Amicon Ultra-0.5 ml Centrifugal filters (EMD Millipore Inc., Billerica, MA, USA). For each sample, a total of 30 μg of protein was reduced with 40 mM DTT, alkylated with 100 mM of iodoacetamide, and trypsin-digested. Trypsin-digested peptides were then desalted with C18-solid phase extraction. A hybrid quadrupole Orbitrap (Q Exactive Plus) mass spectrometry (MS) system (Thermo Fisher Scientific, Bremen, Germany) was used with high energy collision dissociation (HCD) in each MS and MS/MS cycle. The instrument was run in data-dependent mode with a full MS (400–2000 *m*/*z*) resolution of 70 000 and five MS/MS experiments (HCD NCE = 28%, isolation width = 3 Th, first mass = 105 Th, 5% underfill ratio, peptide match set to “preferred”, and an AGC target of 1e6). Dynamic exclusion for 10 s was used to prevent repeated analysis of the same peptides, and a lock mass of *m*/*z* 445.12003 (polysiloxane ion) was used for real-time internal calibration. The MS system was interfaced with an automated Easy-nLC 1000 system (Thermo Fisher Scientific, Bremen, Germany). Each sample fraction was loaded onto an Acclaim Pepmap 100 precolumn (20 mm × 75 μm; 3 μm-C18) and separated on an Easy-Spray analytical column (500 mm × 75 μm; 2 μm-C18) at a flow rate at 300 nL/min during a linear gradient from solvent A (0.1% formic acid (v/v)) to 25% solvent B (0.1% formic acid (v/v), 99.9% acetonitrile (v/v)) for 280 min, followed by ramping up to 98% solvent B for an additional 20 min. Peptides were sprayed into the orifice of the mass spectrometer, which was operated in an information-dependent data acquisition mode.

### Proteomics Data Analysis

Raw MS/MS data files were processed using a thorough database search, considering biological modification and amino acid substitution against a nonredundant *Streptococcus mutans* database with decoy sequences (4,064 entries) using Scaffold Q + S (Proteome Software Inc., Portland, OR, USA) and MASCOT 2.4 (Matrix Science Inc., Boston, MA, USA). The following parameters were used for all the searching: Peptide tolerance at 10 ppm, tandem MS tolerance at ± 0.01 Da, peptide charges of 2+ to 5+ , trypsin as the enzyme, allowing one missed cleavage, Carbamidomethyl (C) as fixed modifications, and oxidation (M) and phosphorylation (S, T, Y) as variable modifications. Peptide and protein were filtered using Scaffold Q + S with strict peptide and protein probabilities, 0.9 and 0.95, respectively. Peptide probability was applied to filter peptide assignments obtained from MS/MS database searching results using predictable false identification error rate. Protein probability was used to filter proteins with the null hypothesis that the database matching is random and consideration of the peptide probability for all the peptides apportioned to that protein. For protein quantification, only MS/MS spectra were normalized by spectral abundance factor (NSAF). To avoid analyzing abundance differences that were unlikely to be biologically relevant, proteins with extremely low abundance (average MS/MS spectral count <2) were excluded from further analysis. Differentially expressed proteins were identified by student *t*-test, and a protein should be quantified with at least three of the biological pentaplicate, and a fold change >1.4 or <0.6 with *p*-value < 0.05. Protein annotation/functional categories presented in all Tables and Supplemental Files were obtained using UniProt^19^, the Los Alamos *S*. *mutans* genome data base (http://www.oralgen.lanl.gov/) and/or the NCBI *S*. *mutans* UA159 reference genome (NC_004350.2). To maximize the number of identified proteins in this analysis, we extensively annotated the detected peptides using the combined outputs of multiple search engines at a stringent FDR.

### Data availability statement

The mass spectrometry proteomics data have been deposited to the ProteomeXchange Consortium via the PRIDE^20^ partner repository with the dataset identifier PXD006735 and 10.6019/PXD006735.

### Correlation analysis with RNA-seq transcriptomic data

To perform correlation analysis with our recently published transcriptomic data^7^, the Reads Per Kilobase of transcript per Million mapped reads (RPKM) were used for all RNA-seq data sets. The *n* = 3 proteomic replicates and triplicate RNA-seq replicates used in the correlation analysis were derived from the same *n* = 3 biological samples. The programming language R was used for all statistical analyses. First, to determine the correlation of the RNA-seq and proteomic datasets, Pearson (linear) correlation was calculated for each two matched samples from RNA-seq and proteomic data using all the genes shared between the two datasets. The expression values were log 2 transformed. The significantly correlated samples were called at thresholds of a false discovery rate (FDR) <0.1. Second, to determine the correlation of the genes between RNA-seq and proteomic datasets across all samples, the Spearman correlation between RNA-seq and proteomic datasets for each gene was calculated using all the samples. The significantly correlated genes were called at thresholds of FDR <0.05 and Spearman’s *rho* >0.5.

## Results and Discussion

We have started to develop an understanding of how Cid and Lrg membrane proteins are related to the ability of *S*. *mutans* to efficiently and rapidly adjust to the ever-changing oral cavity environment, consequently contributing to many key virulence traits of *S*. *mutans* ^5–7^. Since stress responses are closely integrated into bacterial physiology, we also envision that studying the Cid/Lrg system may provide novel insights into the mechanisms that regulate *S*. *mutans* cell homeostasis. The present study provides a valuable opportunity to detect proteomic changes in the same biological set of *S*. *mutans* wild-type and *lrgAB* cultures as those used in our previous transcriptome study^7^, using a label-free mass spectrometry-based quantitative proteomics. The rationale behind this study is that modulating the degradation and/or regulation of proteins may allow the cell to more rapidly and specifically adjust to stress, compared to transcriptomic changes. This more rapid proteome-based response may function to conserve resources by favoring only those cellular processes required for each particular stress condition^21^.

### Overview of changes in protein abundance

**Figure 1:**
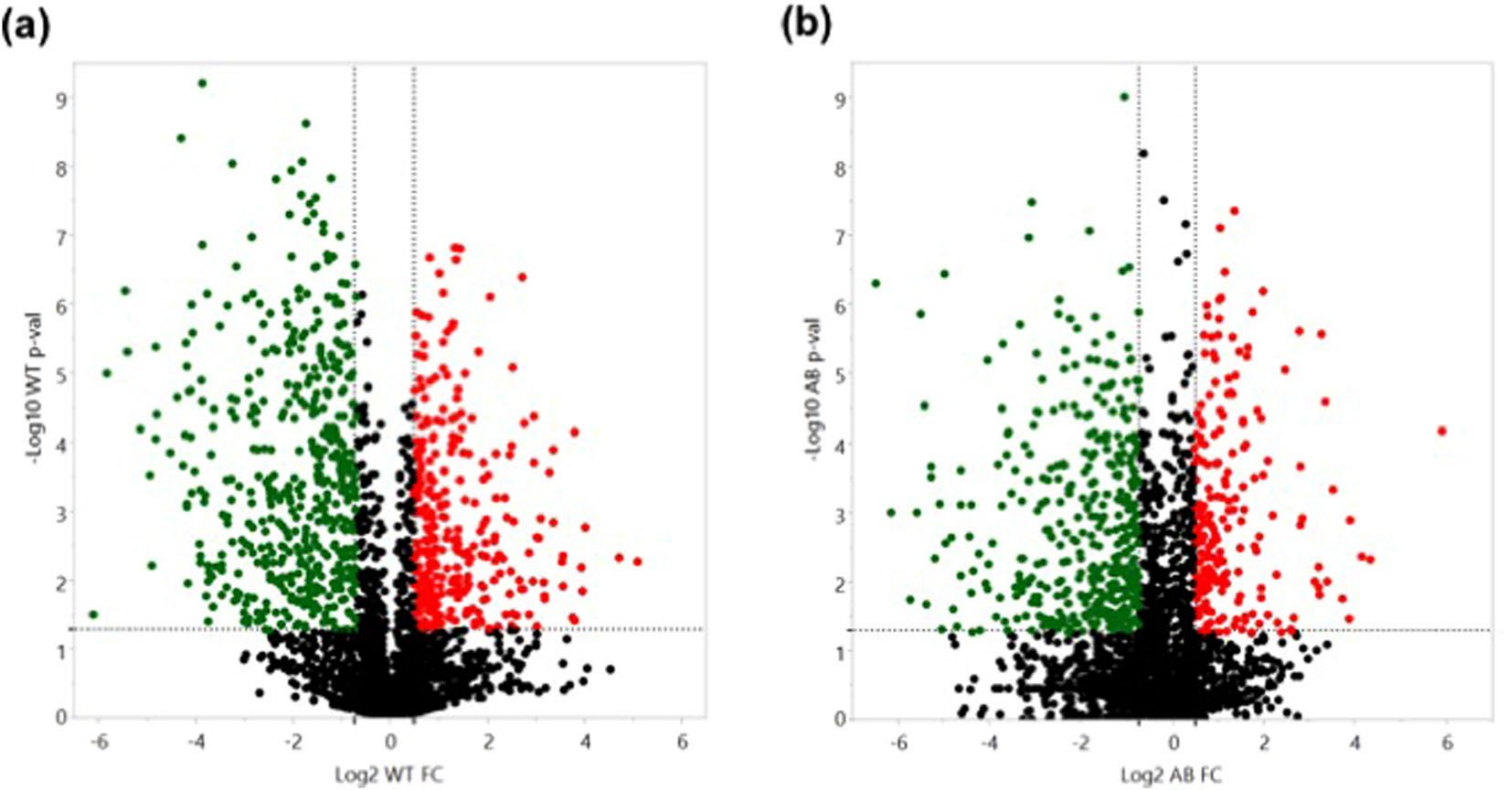
Volcano plots showing differentially accumulated proteins in the wild type (**a**) and *lrgAB* (**b**) strain in response to all three environmental stressors (aeration, heat, and vancomycin stress), relative to non-stress (anaerobic) condition. Cutoff for significantly differential protein abundance, >1.4-fold change in protein abundance and *p*-value < 0.05. Red dots, proteins with increased abundance; green dots, proteins with decreased abundance; black dots, proteins without significant change in abundance. Vertical dotted line, protein abundance cutoff; horizontal dotted line, *p*-value cutoff. WT, wild type strain; AB, *lrgAB* mutant strain.

By using LC-MS/MS and a spectral counting based label-free approach, a total of 1049 proteins in the wild type UA159 were identified at a 95% confidence level, and 535 (51%) of proteins identified were differentially accumulated (a fold change >1.4, or <0.6, *p*-value < 0.05) in response to aeration, heat, or vancomycin stress, compared to the wild-type control samples grown anaerobically (Table 1). In the wild type, more downregulated proteins displayed larger magnitude fold changes as well as higher statistical significance compared to the upregulated proteins (Fig. 1a). In contrast, in the *lrgAB* strain, a total of 1039 proteins were identified at a 95% confidence level, and 474 (46%) of proteins identified were differentially accumulated (a fold change >1.4, or <0.6, *p*-value < 0.05) in response to the same conditions (Table 2). Similar to the wild type strain, more proteins were downregulated with larger magnitude fold changes and higher statistical significance (Fig. 1b). These data suggest that nearly half of the proteins identified by the label-free mass spectrometry differentially responded to the stressful conditions tested in both wild type and *lrgAB* mutant strains. Tables 1 and 2 also summarize the 23 possible protein abundance patterns of *S*. *mutans* wild type (A through W) and *lrgAB* mutant (I through XXIII) strains, respectively, in response to aeration (a), heat (h) and vancomycin (v) stress, relative to control (anaerobic), showing a holistic view for how the proteomes of the organisms shift in response to each stressor or between the stressors. To observe global patterns in the protein abundance data, principal component analysis (PCA) of the complete (both wild-type and *lrgAB* mutant) protein dataset was performed. As shown in Supplemental Fig. S1, the five biological replicates of each strain/growth condition grouped very closely together, except for the anaerobic samples of *lrgAB*. This analysis also suggested that the overall abundance trends in *lrgAB* samples obtained from the aerobic and vancomycin growth conditions were most similar to each other, while the heat *lrgAB* sample cluster was relatively separated from the other *lrgAB* samples. In the wild type strain, each stress sample was greatly separated from the control anaerobic sample cluster. Overall, these data indicate that *S*. *mutans* reorganizes its proteome composition upon exposure to different environmental stressors and possibly through the involvement of LrgAB.

**Table 1:**
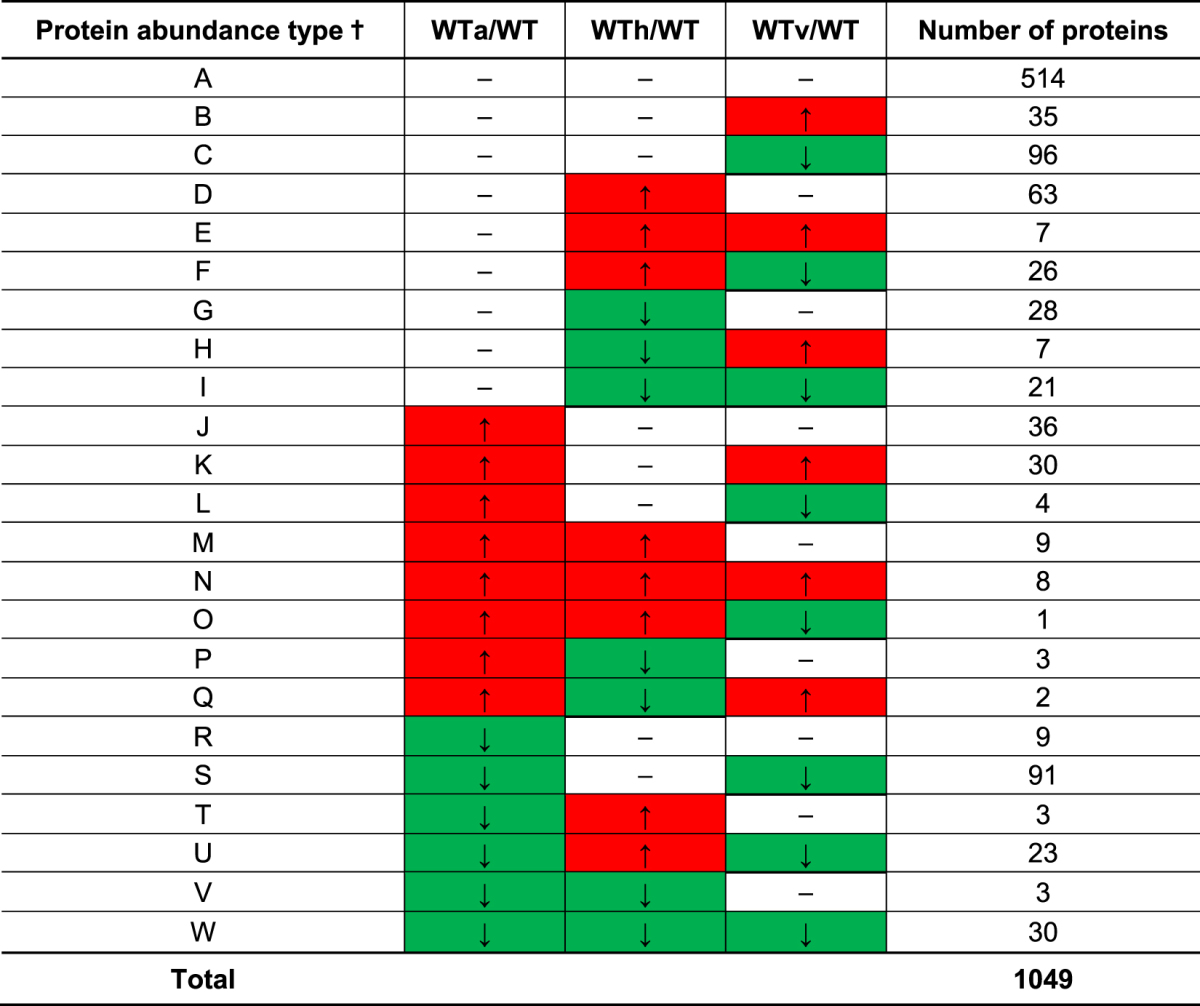
Patterns of differential protein abundance in the wild type in response to environmental stresses, relative to non-stress condition. ^†^The protein abundance patterns of the wild type strain (WT) in response to aeration (a), heat (h) and vancomycin (v) stress, relative to control (anaerobic), to show how the proteomes of the organism shift in response to each stressor or between the stressors; Up-regulated (↑ and red-highlighted), Down-regulated (↓ and green-highlighted).

**Table 2:**
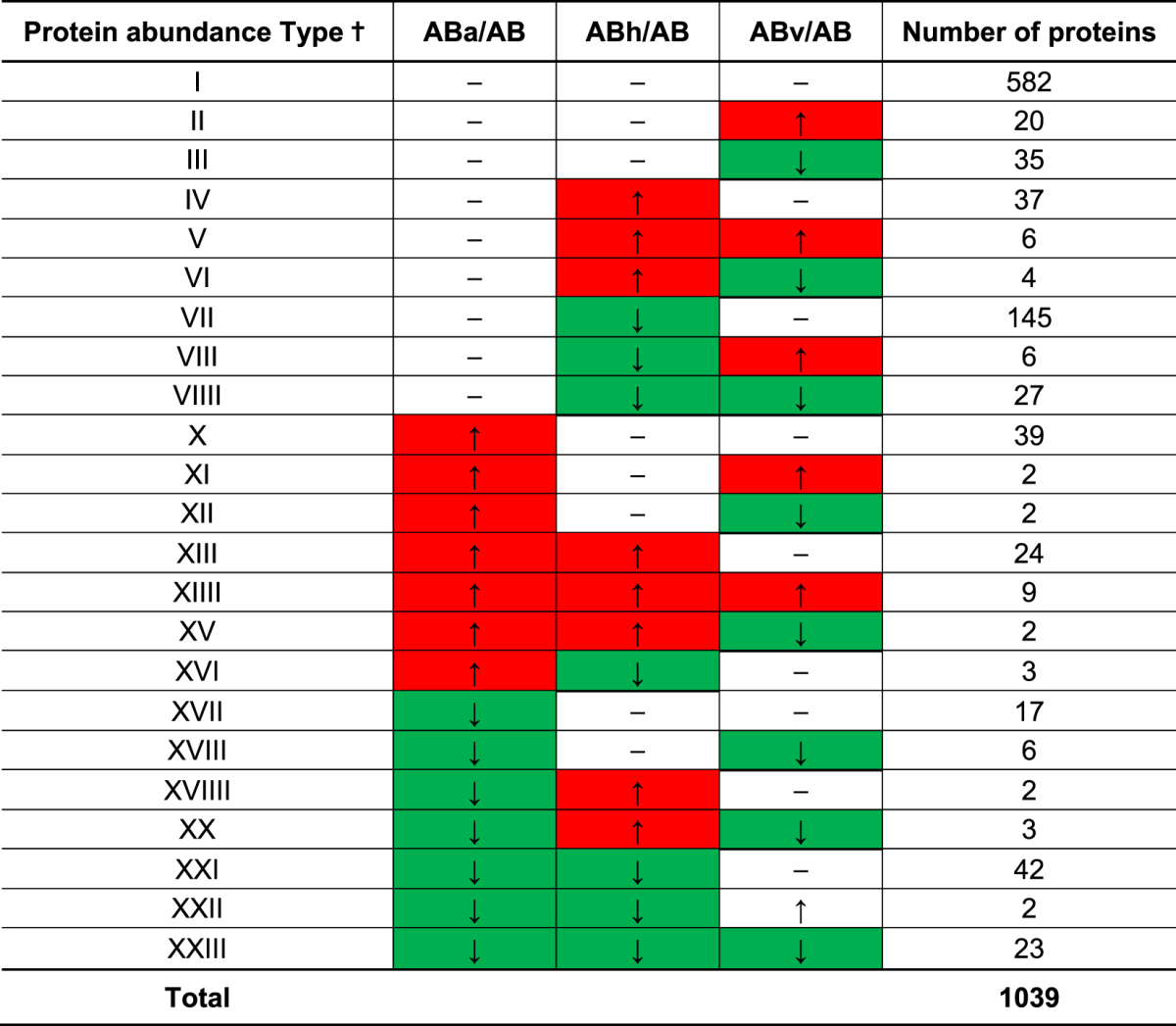
Patterns of differential protein abundance in the *lrgAB* strain in response to environmental stresses, relative to non-stress condition. ^†^The protein abundance patterns of the *lrgAB* mutant strain (AB) in response to aeration (a), heat (h) and vancomycin (v) stress, relative to control (anaerobic), to show how the proteomes of the organism shift in response to each stressor or between the stressors; Up-regulated (↑ and red-highlighted), Down-regulated (↓ and green-highlighted).

### Stress proteomic changes in the wild type strain

**Figure 2:**
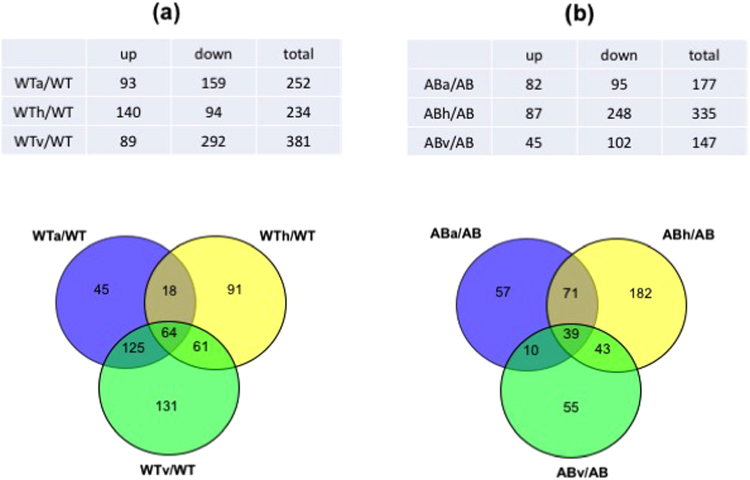
Charts (top) and Venn diagrams (bottom) showing the number of proteins that exhibited differential abundance in the wild type (**a**) and *lrgAB* (**b**) strain in response to aeration (a), heat (h), and vancomycin (v) stress, relative to non-stress (anaerobic) condition. Cutoff for significantly differential protein abundance, > 1.4-fold change in protein abundance and *p*-value < 0.05. WTx, wild type strain in the stress (a, v, or v) condition; WT, wild type strain in the non-stress (anaerobic) condition; ABx, *lrgAB* mutant strain in the stress (a, v, or v) condition; AB, *lrgAB* mutant strain in the non-stress (anaerobic) condition.

The greatest number of proteins (*n* = 381) were differentially accumulated in response to vancomycin stress (Fig. 2a, top), and a considerable number of proteins also changed in abundance when the wild type responded to either aeration (*n* = 252) or heat (*n* = 234), reflecting that roughly 22–36% of the identified *S*. *mutans* proteome was abundantly reorganized to cope with these stressors. A high percentage of the proteins were largely associated with amino acid biosynthesis, energy metabolism, protein synthesis, and transport/binding (Supplemental Table S1), suggesting a substantial reprogramming of bacterial metabolism and physiology in coping with stress. Many proteins that increased or decreased in abundance also had no distinctive function, belonging to hypothetical, unassigned, or unknown categories.

To determine the degree of overlap in protein abundance among all WTx/WT (x = aerobic (a), heat (h) or vancomycin stress (v)), and to better understand the general and specific proteomic responses of the wild-type strain (WT) to each environmental stress, proteins that increased and decreased significantly (*p* < 0.05) in abundance in response to all three stressors were depicted as Venn diagrams (Fig. 2a, bottom). First, significant changes in 64 proteins were common to all three stressors (Fig. 2a, bottom). Among them, more proteins showed decreased (green) expression in response to aeration and vancomycin, while an equal number of proteins showed either an increase (red) or decrease (green) in response to heat (Supplemental Table S2). Notably, the increased amount of HtrA (high-temperature requirement A) in response to all three stress conditions is in line with the primary role of this enzyme in regulation of stress tolerance, mainly against elevated temperature and oxidative stress^22–24^, and virulence^25,26^. 25 proteins had opposite patterns of protein abundance between aeration/vancomycin and high temperature groups. Only one protein (SMU_1090; uncharacterized protein) was upregulated in response to both aeration and heat, but downregulated to vancomycin stress. Thus, unlike the response to aeration and vancomycin, response to heat may involve the contribution of additional or different physiological mechanisms in the wild type strain. The common 64 proteins were assigned to 16 different functional categories and alterations in protein abundance were enriched in the categories of amino acid biosynthesis (*n* = 10) and transport/binding proteins (*n* = 9) (Supplemental Table S2), suggesting that these functions are altered in *S*. *mutans* as an adaptive response to environmental stress.

We also identified protein signatures representing unique responses to each stress in the wild type (*n* = 45 for aeration; *n* = 91 for heat stress; *n* = 131 for vancomycin; Fig. 2a, bottom). Notable protein abundance changes unique to aeration include upregulated accumulation of putative chorismate mutase (SMU_531), cell division protein FtsX, and putative mannose specific EIID component (SMU_1957), which were newly produced only in response to aeration, but not without aeration (Supplemental Table S3). It is also noteworthy that SMU_1904c (uncharacterized protein), previously shown to be one of the most oxygen-sensitive genes^7,27^, was also more than 15-fold upregulated at the protein level, reinforcing the role of this gene/protein in coping with oxidative stress. Other highly upregulated proteins included GshR (glutathione reductase) and CopZ (putative copper chaperone), which were more than 8-fold and 4-fold upregulated, respectively, and have been previously reported to be important in protection of *S*. *mutans* against oxidative stress^28,29^. Particularly, CopZ has also been shown to be important in modulating a variety of virulence traits, including biofilm formation, stress tolerance, genetic competence, and competitiveness against commensal streptococci^30^. In contrast, the production of two putative ABC transporters (SMU_238c and SMU_370) and possible transmembrane efflux protein (SMU_1605) was completely suppressed in response to aeration (Supplemental Table S3). Interestingly, no downregulated proteins uniquely responding to aeration were differentially expressed at the transcriptional level, except for SMU_1340 (putative surfactin/bacitracin synthetase)^7^, which is part of TnSmu2, shown to enhance aerobic growth and tolerance to H_2_O_2_ challenge in *S*. *mutans* through nonribosomal peptide and polyketide (NRP/PK) biosynthesis^31^. Next, it is noteworthy that proteins uniquely responding to heat in the wild type, including multiple transcriptional (CpsY, Rex, NrdR, SMU_1398, SMU_677, and SMU_1585c) and global (CcpA and FruR) regulators, suggesting that response to heat stress may require a tighter coordination of both gene and protein expression and/or modification, probably to avoid unwanted cellular and physiological changes (Supplemental Table S4). Multiple ABC transporters, including OpuBa, SMU_922, SMU_1194, and SMU_1568 also showed significantly (*p* < 0.05) increased abundance. Major functional categories that were uniquely upregulated in response to heat stress included energy metabolism and PTSs, whereas DNA metabolism and protein synthesis-related proteins showed significantly decreased abundance, suggesting possible metabolic rewiring for production of extra energy (ATP) to cope with this stress^32,33^. It is also notable that among the heat-shock related molecular chaperones and proteases, only DnaK and Clp-like proteases (SMU_956 and SMU_2029) showed increased abundance uniquely in response to heat stress, suggesting that these proteins may be especially important for *S*. *mutans* cells living in environments with continuous heat. Interestingly, MazF, the predicted toxin component of the MazEF toxin/antitoxin (TA) module, was newly produced and uniquely responded to heat stress. This likely contributes to cell vialbility during heat stress, as overexpression of MazF presumably results in growth inhibition^34^. In contrast, the production of Cas9 (SMU_1405c), a CRISPR-associated endonuclease, was completely suppressed (Supplemental Table S4). Although how Cas proteins are involved in the response to heat stress is unclear, mutations in CRISPR1-*cas* and CRISPR2-*cas* in *S*. *mutans* were both previously shown to confer increased sensitivity to heat stress^35^. Overall, these data suggest that the response to heat stress in the *S*. *mutans* wild type strain may be regulated by a greater number of cellular processes than previously thought. Protein abundance changes unique to vancomycin stress occurred in multiple uncharacterized proteins and ABC transporters (Supplemental Table S5). In fact, tolerance to various peptide antibiotics, including vancomycin, has been reported to coevolve with ABC transporters and neighboring TCSs^36^. It is also notable that a great number of proteins involved in translation showed significantly decreased abundance. Given that vancomycin targets cell wall biogenesis and metabolism, it is not surprising that proteins involved in these processes were upregulated in response to this stress, including GtfC, WapA, WapE, AtlA (SMU_689), DacA and PtsH.

Finally, Fig. 2a (bottom), also showed that many proteome changes overlapped between two stressors. The largest overlapped proteins (*n* = 125) were observed between aeration and vancomycin proteome changes, while the smallest overlapped proteins (*n* = 18) were observed between aeration and heat proteome changes. Overall, it is notable that the proteome changes of wild-type were quite different from its transcriptome changes^7^ in response to the three stressors (Fig. 2a). In particular, *S*. *mutans* responded to vancomycin primarily through proteome readjustment, as very few changes were observed at the transcriptional level^7^. In contrast, the response of the organism to heat was more profound at the transcriptional level (*n* = 425)^7^, compared to its corresponding proteomic changes (*n* = 234). Aeration moderately influenced both the proteome and transcriptome of the organism. Therefore, these data suggest that proteome adjustment may provide an alternative way to respond to stress as a counterpoint to transcriptional modulation, and be intertwined with expression of virulence-related molecular elements.

### Stress proteomic changes in the *lrgAB* mutant strain

Unlike the wild type strain, the proteome changes of the *lrgAB* mutant were most affected by heat (*n* = 335), and least by vancomycin stress (*n* = 147) (Fig. 2b, top). 177 proteins were also altered in abundance in the *lrgAB* mutant when cultured aerobically (Fig. 2b, top). Overall, stress responsive proteins corresponded to 14–32% of the identified *lrgAB* mutant proteome (*n* = 1039) (Table 2). Notably, except for the uncharacterized proteins (belonging to “hypothetical”, “unassigned” or “unknown” categories), many proteins associated with amino acid biosynthesis were consistently downregulated in response to all three stresses (Supplemental Table S6). Another major functional category with substantial changes in protein abundance was energy metabolism, which was largely upregulated in response to aeration, and downregulated to heat and vancomycin stress. However, the functional alteration profile was similar to that of the wild type in response to these stresses.

As shown in a Venn diagram displaying the degree of overlap in protein abundance among all ABx/AB (Fig. 2b; bottom), 39 proteins showed significant (*p* < 0.05) changes in common under all three stressors. Unlike the wild type, the *lrgAB* mutant showed relatively similar protein abundance patterns in response to all three stressors, with the exception of 7 proteins (Supplemental Table S7). Among the common 39 proteins, 11 proteins, including GbpA, SMU_218 (putative transcriptional regulator), Ddl (SMU_599), Obg (SMU_801), MsmK, SecA, SMU_1717c, Pnp (SMU_155), SMU_1416c, SerB, and HisD, were also contained in the overlapping set of proteins in the wild type (Supplemental Tables S7), which could be a potential core stress regulon common to all three stressors in *S*. *mutans*. Other notable upregulated proteins included Ftf, SMU_609 (putative 40 K cell wall protein), SMU_1287 (putative transcriptional regulator) and SMU_769. Particualrly, SMU_769 is one of the late competence genes regulated by ComX (or SigX), an alternative sigma factor of *S*. *mutans*.^37,38^. Other notable downregulated proteins included AtlA, SerB, DexA, amd SMU_1661c (putative signal peptidase). Interestingly, two putative cell wall hydrolases, SMU_609 and AtlA, responded to all three stresses in an opposite manner in the *lrgAB* strain (Supplemental Table S7), suggesting different role of the putative hydrolases in cell wall turnover and stress tolerance. The most obvious alterations in protein abundance belonged to amino acid biosynthesis (*n* = 6) and energy metabolism (*n* = 6).

In the *lrgAB* mutant, the greatest number of proteins (*n* = 182) uniquely responded to heat (Fig. 2b; bottom), and the majority of the proteins (*n* = 145) showed a significant decrease in abundance (Supplemental Table S8). Particularly, production of 25 proteins unique to this stress was completely suppressed in the *lrgAB* strain, and over 40 proteins were more than 5-fold downregulated, suggesting that adaptation to heat may require radical proteome turnover. Notable downregulated proteins included CovX, RelR, ScrK, GbpD, Pbp2b, RecR, RecX, SloR, and ComR (Supplemental Table S8). Given that ComR is a proximal regulator of ComX (SigX)^39^, decreased genetic competence of the *lrgA* strain may be related to altered ComR levels^4^. Proteins with increased abundance showed relatively lower fold-changes and included PtsH, SpxA1, FruI, GbpC, MazE, WapA, CiaR, DnaJ and GrpE. In the *lrgAB* mutant strain, interestingly, abundance of MazE, the putative antitoxin protein of the MAzEF T/A module, was increased, whereas MazF showed increased abundance in the wild type in response to heat (Supplemental Table S5). This suggests that MazEF, a proposed mediator of bacterial programmed cell death^40,41^, is closely linked to the heat stress response, as well as LrgAB. Another notable protein is SpxA1, which was previously shown to play an important role in stress tolerance, survival, and virulence in *S*. *mutans* with SpxA2^42,43^. However, interestingly, SpxA2 did not significantly respond to any stress in both wild type and *lrgAB*-deficient strains, supporting a more dominant role of SpxA1 than SpxA2^43^. Similar numbers of proteins uniquely responded to aeration (*n* = 57) and vancomycin stress (*n* = 55), which are shown in Supplemental Tables S9 and S10, respectively. Seven uncharacterized proteins were newly produced or >10-fold upregulated uniquely in response to aeration, while the downregulated proteins showed a relatively smaller fold-change (Supplemental Table S9). It is interesting that the production of SMU_571, located downstresm of *lrgAB*, was completely suppressed in the *lrgAB* strain uniquely in response to aeration and forms a three gene-operon with two putative ferrous iron transport proteins (FeoA/B, SMU_569/570). Given that iron plays a role in protection against oxidative stress^44,45^, SMU_571 may contribute to enhanced sensitivity of *lrgAB* to aeration, possiblly as a signaling peptide associated with FeoA/B transporters. It is also noteworthy that MvaK, a putative mevalonate kinase, was newly produced only when the *lrgAB* mutant responded to aeration (Supplemental Table S9), suggesting a possible functional linkage of LrgAB with the mevalonate pathway, which is involved in a variety of vital biological functions in bacteria, including cell wall biosynthesis^46,47^. In response to vancomycin stress, the fold-change of upregulated proteins (largely ribosomal proteins) in the *lrgAB* mutant was moderate, while downregulated proteins showed greater fold-change or were not accumulated (Supplemental Table S10). Notable proteins with decreased abundance include RcrR (SMU_921), PknB, GtfB, and MazF, which are related to virulence traits such as genetic competence, surface attachment, and stress response of *S*. *mutans* ^34,48–52^. Overall, these results suggest that the absence of LrgAB elicits extensive rewiring of the *S*. *mutans* proteome, particularly related to stress responses and virulence traits.

### Comparison of UA159 and Δ*lrgAB* proteome changes in response to each stressor

**Figure 3:**
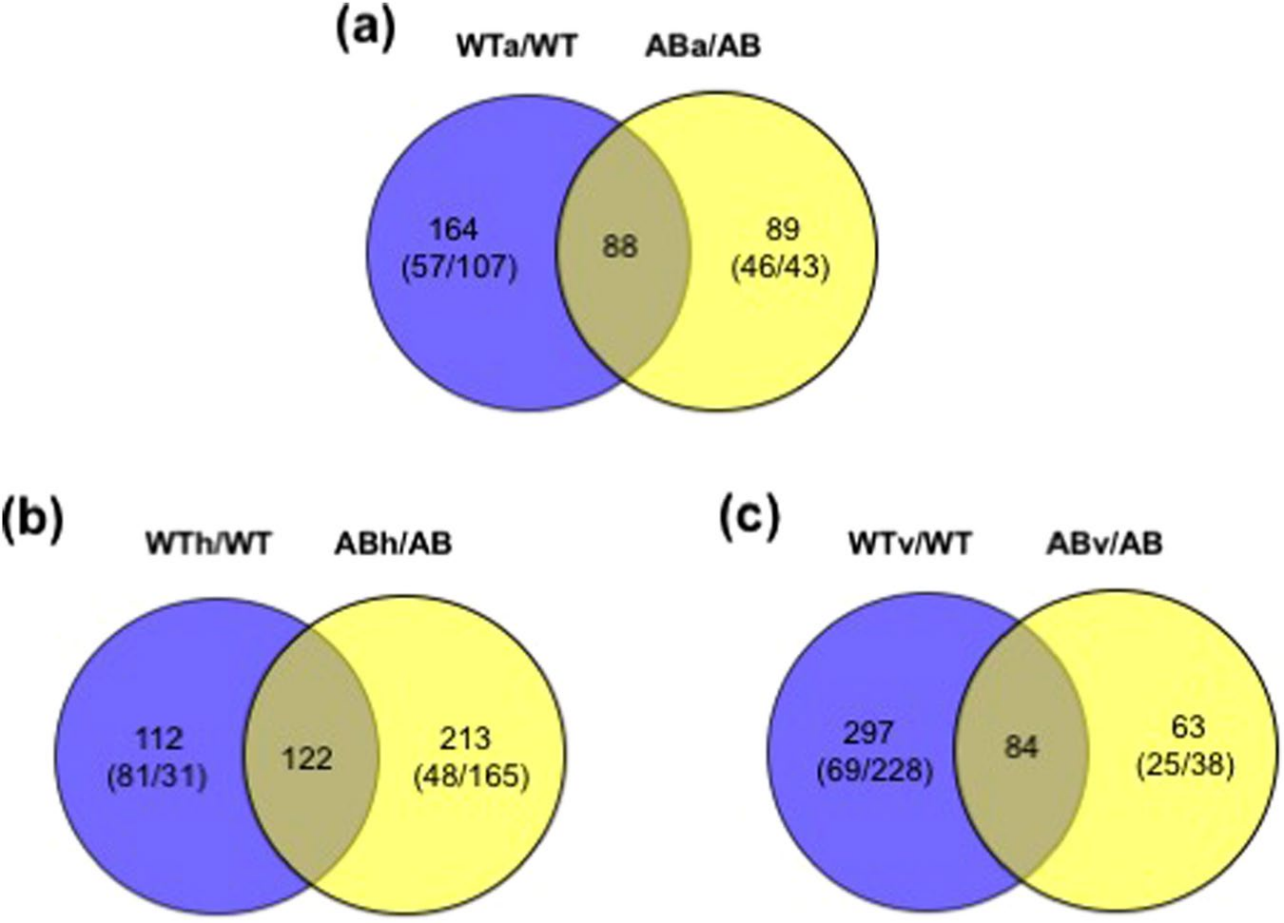
Venn diagrams showing the comparison of proteome changes of wild type (WTx/WT) and *lrgAB* (ABx/AB) in response to aeration (**a**), heat (**b**), and vancomycin (**c**) stress. Cutoff for significantly differential protein abundance, >1.4-fold change in protein abundance and *p*-value < 0.05. WTx, wild type strain in the stress (a, v, or v) condition; WT, wild type strain in the non-stress (anaerobic) condition; ABx, *lrgAB* mutant strain in the stress (a, v, or v) condition; AB, *lrgAB* mutant strain in the non-stress (anaerobic) condition. The numbers in parenthesis indicate (up/down)-regulated proteins.

To better understand how LrgAB is involved in coping with each stress, we compared the proteome changes of wild type (WTx/WT) and *lrgAB* (ABx/AB) in response to each stressor with a focus on the *lrgAB-*specific proteome changes, using Venn diagrams (Fig. 3). In response to aeration, 88 proteins demonstrated substantial changes in common between the WTa/WT and ABa/AB proteomes (Fig. 3a; Supplemental Fig. S2; Supplemental Table S11), representing the *S*. *mutans* general response to aerobic growth. One interesting finding is that two uncharacterized proteins (SMU_1209c and SMU_2152c) showed an opposite abundance pattern between the wild type and *lrgAB* mutant in response to aeration. 89 proteins responded to aeration only in the *lrgAB* strain, one or more of which may be responsible for the growth defect of the strain under the aerobic condition (Fig. 3a, Supplemental Fig. S2, Supplemental Table S12). 122 proteins showed significant (*p* < 0.05) abundance changes common to both WTh/WT and ABh/AB proteome comparisons (Fig. 3b, Supplemental Fig. S3, Supplemental Table S13). 20 proteins showed an opposite abundance pattern between wild type and *lrgAB* mutant in response to heat stress, and all of them were upregulated in the wild type and downregulated in the *lrgAB* strain. Among them, notable proteins showing increased abundance by more than 3-fold in the wild type included CpsY, TrpB, MurD, PyrK, SMU_1194 (putative ABC transporter), and SMU_521 (uncharacterized protein). Particularly, CpsY, a conserved transcriptional regulator, is known to activate transcription of genes involved in methionine biosynthesis and uptake in *S*. *mutans* ^53^ and *S*. *agalactiae* ^54^. CpsY has been also characterized as as virulence determinant that regulates amino acid metabolism^54^, methionine transport^55^, and cell wall modifications necessary for systemic infection in *S*. *iniae* ^56–58^. Therefore, CpsY may play a role in the Cid/Lrg system that relates the environmental/stress status of *S*. *mutans* to the control of growth and expression of virulence traits. A great number of proteins (*n* = 213) differentially responded to heat in the *lrgAB* mutant only (Fig. 3b, Supplemental Fig. S3, Supplemental Table S14). More proteins (*n* = 165) showed a significantly (*p* < 0.05) decreased abundance, and among them 82 proteins showed a fold change by more than 5-fold or were not accumulated at all. SloR, a DtxR family metalloregulator, known to modulate *S*. *mutans* metal ion homeostasis, biofilm formation, oxidative stress tolerance, and antibiotic gene regulation^59–64^, was uniquely suppressed in the *lrgAB* mutant in response to heat stress. SloR was also recently shown to crosstalk with VicKR and GcrR, major regulatory systems controlling critical *S*. *mutans* cellular processes and virulence^65–69^, through manganese availability^60,70^. Since VicKR was shown to regulate expression of *lrgAB* ^5^, SloR may be one of the intimate regulators collaborating with LrgAB. Lastly, 84 proteins demonstrated significant (*p* < 0.05) changes common to both WTv/WT and ABv/AB proteomes (Fig. 3a, Supplemental Fig. S4, Supplemental Table S15). More proteins were downregulated (*n* = 64) than upregulated (*n* = 20), and among them twelve proteins showed an opposite abundance pattern between wild type and *lrgAB* mutant in response to vancomycin stress. Interestingly, one uncharacterized protein (SMU_843) was newly induced in the wild type, and not accumulated in the *lrgAB* strain. This protein is predicted to play a role in cell wall/envelope biosynthesis as a putative poly-gamma-glutamate biosynthesis protein. In response to vancomycin stress, less proteome abundance changes were uniquely elicited in the *lrgAB* strain (*n* = 63) than wild-type (*n* = 297) (Fig. 3c, Supplemental Fig. S4, Supplemental Table S16). One interesting finding is that accumulation of GtfB, a key adhesin of *S*. *mutans*, was completely inhibited in the *lrgAB* mutant in response to vancomycin stress, supporting the potential for LrgAB as an anti-biofilm target.

### Proteome changes between wild type and *lrgAB* strains during non-stressed growth

When we directly compared the proteome of wild-type and *lrgAB* mutant in an optimal growth condition (anaerobic; at 37 °C), loss of LrgAB significantly (*p* < 0.05) altered the expression of 76 proteins (a fold change >1.4, or <0.6, *p*-value < 0.05) with 17 downregulated and 59 upregulated (Supplemental Fig. S5a; Table 3). While the previously-published corresponding RNA-seq analysis showed that most of the 54 differentially-expressed genes belonged to very few distinct functional groups (genomic islands (GIs) TnSmu1 and TnSmu2, the CRISPR (clustered regularly interspaced short palindromic repeats)-Cas system, bacteriocin production, energy metabolism, and amino acid ABC transporters)^7^, altered proteins belonged to many more diverse functional categories, including cell envelope, cellular processes, regulatory functions, and signal transduction (Supplemental Fig. S5b; Table 3). Nevertheless, SMU_209 (down-regulated) and SMU_1348c (up-regulated), overlapped between the transcriptome and proteome profiles, belonged to TnSmu1 and TnSmu2, respectively, which were two major functional groups with most differentially expression genes in the transcriptome profile, reinforcing the involvement of these genomic islands with the Cid/Lrg system. Other notable upregulated proteins included two putative transcriptional regulators (CpsY and SMU_1977c), two fatty acid biosynthesis-related proteins (AccC and FabZ), MazF, NaoX (H_2_O-forming NADH oxidase), GtfC, and GlgC, which were newly induced or had a fold-change by more than 4-fold. These data suggest that mutation of *lrgAB* may impose inherent stress on *S*. *mutans* cells, as well as shows potential functional partners of LrgAB and related molecular processes.

**Table 3:**
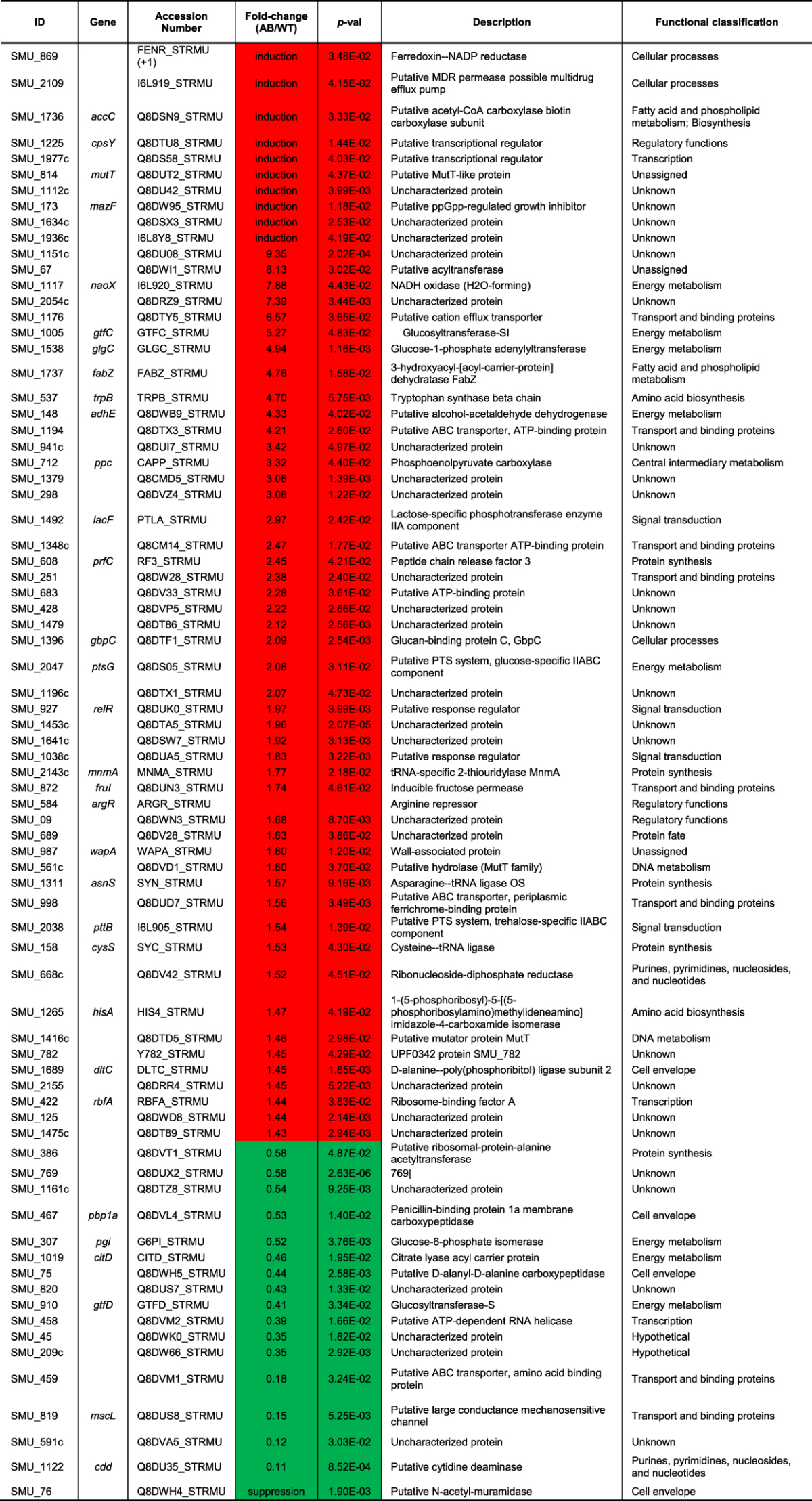
Altered protein abundance between wild-type and *lrgAB* mutant during anaerobic (unstressed) growth. ^†^Fold-change: ‘red’-highlighted, upregulated; ‘green’-highlighted, downregulated; ‘induction’, newly accumulated; ‘suppression’, not accumulated.

### Correlation with RNA-seq transcriptomic profiles

We attempted to determine the relationship between the proteomic profiles and the gene expression profiles, recently obtained by high-throughput RNA-seq technology on corresponding aliquots of the same cultures used for this proteome experimentation^7^. As the transcriptome datasets contain three biological replicates per condition, only the three matched samples from each condition were used. In other words, correlation analysis was performed on a total of 24 samples from all conditions for RNA-seq and proteomic datasets, respectively. First, Pearson correlation was performed for the 24 matched samples to assess sample level correlation using all the shared genes/proteins for the matched RNA-seq and proteomic datasets. All of the 24 samples had a relative low positive correlation coefficient (*rho*(ρ) <0.16) between the RNA-seq and proteome datasets (Supplemental Table S17). Although this correlation was not high, the correlation coefficients for the 15 datasets representing AB, ABa, ABv, WT and WTh samples (in triplicates) are statistically significant (*p* < 0.05) with *q-*values (multiple testing corrected *p-*value; FDR) less than 10% (Supplemental Table S17). Therefore, the proteome changes that occur in the *lrgAB* mutant in response to heat stress, and in the wild-type in response to aeration and vancomycin stress, may be potentially elicited by non-transcriptional mechanisms such as mRNA decay, translation, and protein degradation and modification. In order to find the specific genes/proteins that contributed to the correlation between the transcriptome and proteome datasets, we also performed Spearman (monotonic) correlation analysis across all samples. At thresholds of *q-*value 0.05 and *rho* >0.5, a total of 46 genes (~6%) out of 776 tested genes showed significant (*p* < 0.05) correlation. Of these, 42 represent significantly differentially-expressed proteins (Supplemental Table S18). We also counted the number of differentially-expressed genes that overlapped between the RNA-seq and proteome datasets, and found that only a small proportion of these genes ( < 9%) were overlapped (Supplemental Table S19). The small proportion of correlated genes is consistent with the low sample level correlation results described above. Possibly, the low correlaton between transcriptomic and proteomic changes in this study may be due to continuous growth of the bacteria in the presence of each stress; this may have facilitated degradation or modification of damaged or unfolded proteins, to avoid deleterious consequences such as cell death or growth inhibition^71,72^. Another factor that may have lowered our ability to detect a higher correlation is that the transcriptome and proteome datasets were substantially different in size. Our comparison between transcriptome and proteome profiles was limited to the genes for which corresponding proteins were identified by mass spectrometry. The size difference was even bigger per stress condition^7^. Therefore, gaps in our proteomic dataset could have led to a comparison of a biased selection of only highly abundant proteins with unbiased expression data. A third factor may be the limited number of samples used for the correlation analysis. We did correlation analysis for each gene/protein using the three biological samples within each condition. In this case, perfect correlation were identified *(rho* = 1) in 707 genes/proteins but none is statistically significant (*P* value > 0.3). In contrast, when used the 24 biological samples across all the conditions, we identified 46 significantly (*p* < 0.05) correlated genes/proteins. Therefore, it is possible that with more biological samples, more significantly correlated genes can be potentially identified. Finally, from a technical standpoint, the approach using mass spectrometry would not completely cover the identification of rather hydrophobic proteins, although several cell membrane and surface proteins were identified here. Also, the current experimental approach did not not allow the quantitative analysis of secreted proteins, consequently generating a less representative proteome than its corresponsing transcriptome.

In summary, to our knowledge, this is the first comparative study on the intracellular proteome of *S*. *mutans* under multiple stress conditions. The data suggest that *S*. *mutans* adjusts its stress response through substantial and dynamic remodeling of the cellular proteome, and LrgAB has an important influence on this reorganization. This study also provided a valuable opportunity to compare transcriptomic and proteomic data generated on corresponding aliquots of the same cultures. It is possible that the overall low correlation between two ‘-omics’ datasets may be due to potential analytical and technical limitations, as well as unknown cellular mechanisms in the presence of rapid or continuous stress shock. These two integrative omics studies have provided a large number of transcripts and proteins that could be used in targeted studies on the molecular mechanisms triggering the persistence phenotype of *S*. *mutans* against unfavorable stresses. The data may also give an integrative view of a specific physiological state in coping with an external stress. More detailed biological interpretation of the altered proteins will be an interesting future direction. Given that within biofilms, oral bacteria become more resistant to adverse environments, including antibiotic challenge, compared to planktonic growth, it would be interesting to evaluate the proteomic changes elicited by these environmental stresses on *S*. *mutans* biofilms. This approach could provide more ecological insights into how this bacterium accommodates the heterogeneous microenvironments that develop in biofilm, as well as accelerate phenotypic and genotypic diversity within the population. Furthermore, studying proteomic shifts in multispecies oral biofilms by *S*. *mutans* in response to other types of environmental challenges, such as nutrient limitation and toxic metabolites, could be another interesting follow-up. Consequently, all these data will improve our integrated models and understanding of how bacterial cells adjust to a new challenging environment, an initial step in initiation of disease.

## Electronic supplementary material

Supplemental Figures

Supplemental Dataset 1
