# Supplementary material for: Remodeling of the Streptococcus mutans proteome in response to LrgAB and external stresses
Source: Sci Rep. 2017 Oct 25;7:14063. doi: 10.1038/s41598-017-14324-w (PMC5656683; doi:10.1038/s41598-017-14324-w)
Supplement: Supplementary file 1 — Supplemental Figures [file 41598_2017_14324_MOESM1_ESM.pdf]

Supplementary Figures For:

Remodeling of the *Streptococcus mutans* proteome in response to LrgAB and external stresses

Sang-Joon Ahn[1Ψ], Tongjun Gu[2], Jin Koh[3], and Kelly C. Rice[4]

[1]Department of Oral Biology, College of Dentistry, University of Florida, Gainesville, FL 32610, USA

[2]Bioinformatics, Interdisciplinary Center for Biotechnology Research, University of Florida, Gainesville, FL32610, USA

[3]Proteomics and Mass Spectrometry, Interdisciplinary Center for Biotechnology Research, University of Florida, Gainesville, FL32610, USA

[4]Department of Microbiology and Cell Science, Institute of Food and Agricultural Sciences, University of Florida, Gainesville, FL 32611, USA

[Ψ]Corresponding author:

Mailing address: Department of Oral Biology, University of Florida, College of Dentistry, P.O. Box 100424, Gainesville, FL 32610

Telephone: (352) 273-8834

Fax: (352) 273-8829

Email: sahn@dental.ufl.edu

Supplemental figures:

Fig. S1. PCA of proteomic data. All identified proteins were analyzed by Principle Components Analysis (PCA), as described in Materials and Methods

Fig. S2. Functional categorization of the proteins altered commonly to both wild-type and *lrgAB* (*n*=88), and uniquely to *lrgAB* (*n*=89), respectively, in response to aeration (a fold change >1.4, or <0.6, *p*-value <0.05). The proteins are grouped by functional classification according to the Los Alamos *S. mutans* genome database (http://www.oralgen.lanl.gov/).

Fig. S3. Functional categorization of the proteins altered commonly to both wild-type and *lrgAB* (*n*=122), and uniquely to *lrgAB* (*n*=213), respectively, in response to aeration (a fold change >1.4, or <0.6, *p*-value <0.05). The proteins are grouped by functional classification according to the Los Alamos *S. mutans* genome database (http://www.oralgen.lanl.gov/).

Fig. S4. Functional categorization of the proteins altered commonly to both wild-type and *lrgAB* (*n*=84), and uniquely to *lrgAB* (*n*=63), respectively, in response to aeration (a fold change >1.4, or <0.6, *p*-value <0.05). The proteins are grouped by functional classification according to the Los Alamos *S. mutans* genome database (http://www.oralgen.lanl.gov/).

Fig. S5(a). Volcano plots showing differentially accumulated proteins in the *lrgAB* relative to wild type during unstressed growth. Cutoff for significantly differential protein abundance, >1.4-fold change in protein abundance and *p*-value <0.05. Red dots, proteins with increased abundance; green dots, proteins with decreased abundance; black dots, proteins without significant change in abundance. Vertical dotted line, protein abundance cutoff; horizontal dotted line, *p*-value cutoff. WT, wild type strain; AB, *lrgAB* mutant strain.

Fig. S5(b). Distribution of 76 proteins with a significant ($p < 0.05$) **change in abundance in the *lrgAB* relative to wild type during unstressed growth, based on predicted function.**

Supple. Fig. S1

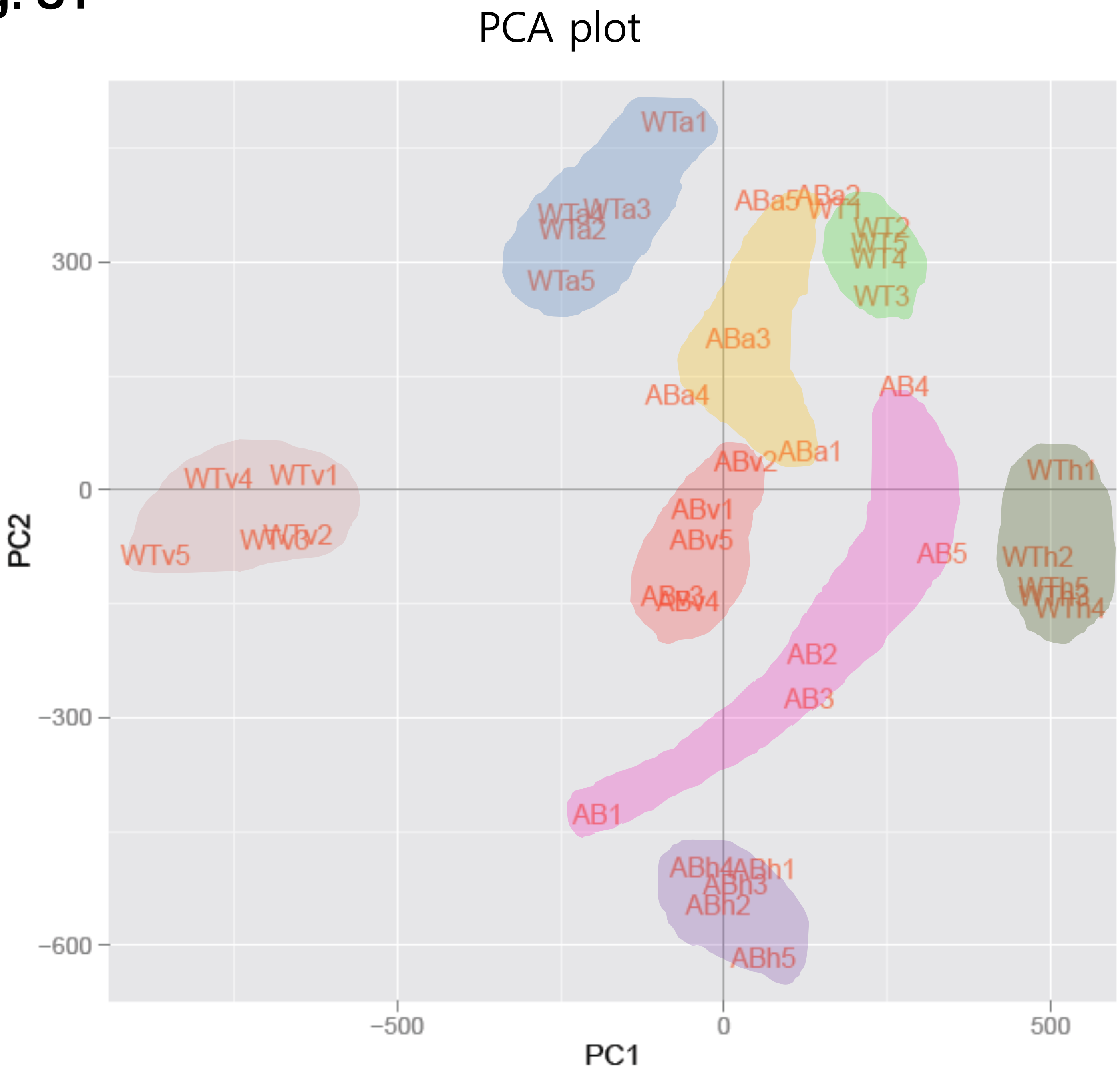

Fig. S2

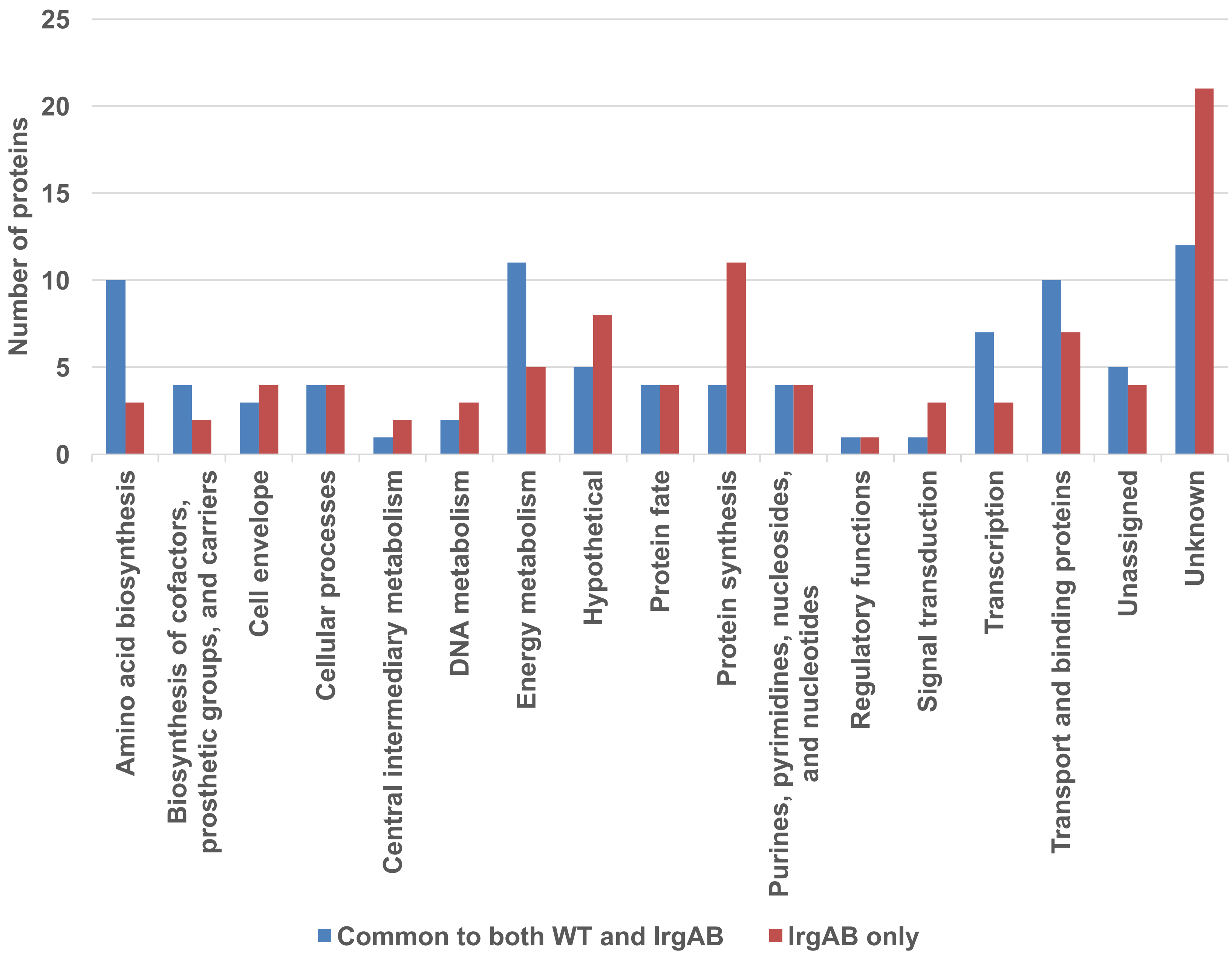
25
20
15
10
5
0
Number of proteins
Amino acid biosynthesis
Biosynthesis of cofactors, prosthetic groups, and carriers
Cell envelope
Cellular processes
Central intermediary metabolism
DNA metabolism
Energy metabolism
Hypothetical
Protein fate
Protein synthesis
Purines, pyrimidines, nucleosides, and nucleotides
Regulatory functions
Signal transduction
Transcription
Transport and binding proteins
Unassigned
Unknown
Common to both WT and lrgAB
lrgAB only

Fig. S3

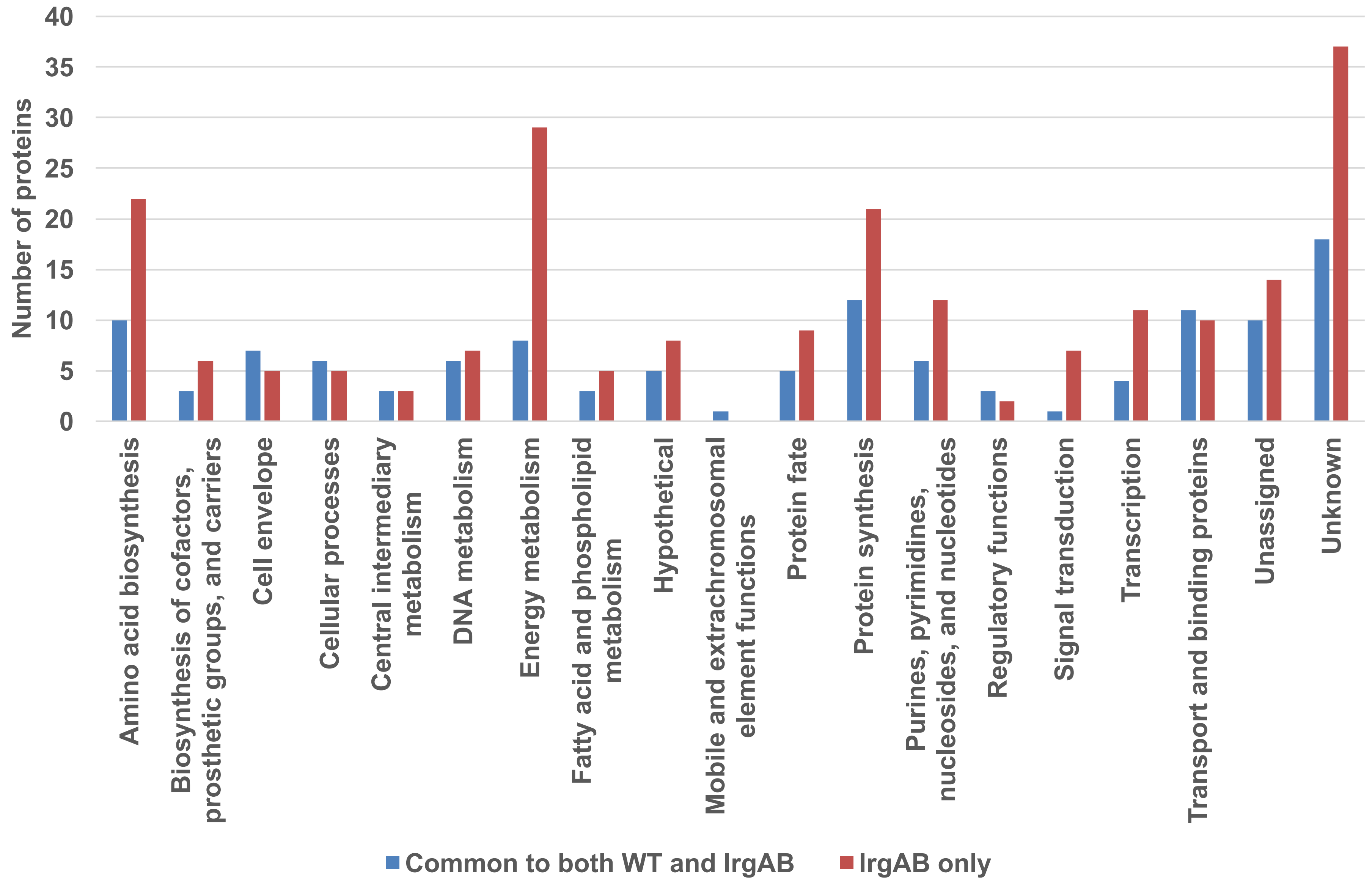

Number of proteins
40
35
30
25
20
15
10
5
0
Amino acid biosynthesis
Biosynthesis of cofactors, prosthetic groups, and carriers
Cell envelope
Cellular processes
Central intermediary metabolism
DNA metabolism
Energy metabolism
Fatty acid and phospholipid metabolism
Hypothetical
Mobile and extrachromosomal element functions
Protein fate
Protein synthesis
Purines, pyrimidines, nucleosides, and nucleotides
Regulatory functions
Signal transduction
Transcription
Transport and binding proteins
Unassigned
Unknown
Common to both WT and lrgAB
lrgAB only

Fig. S4

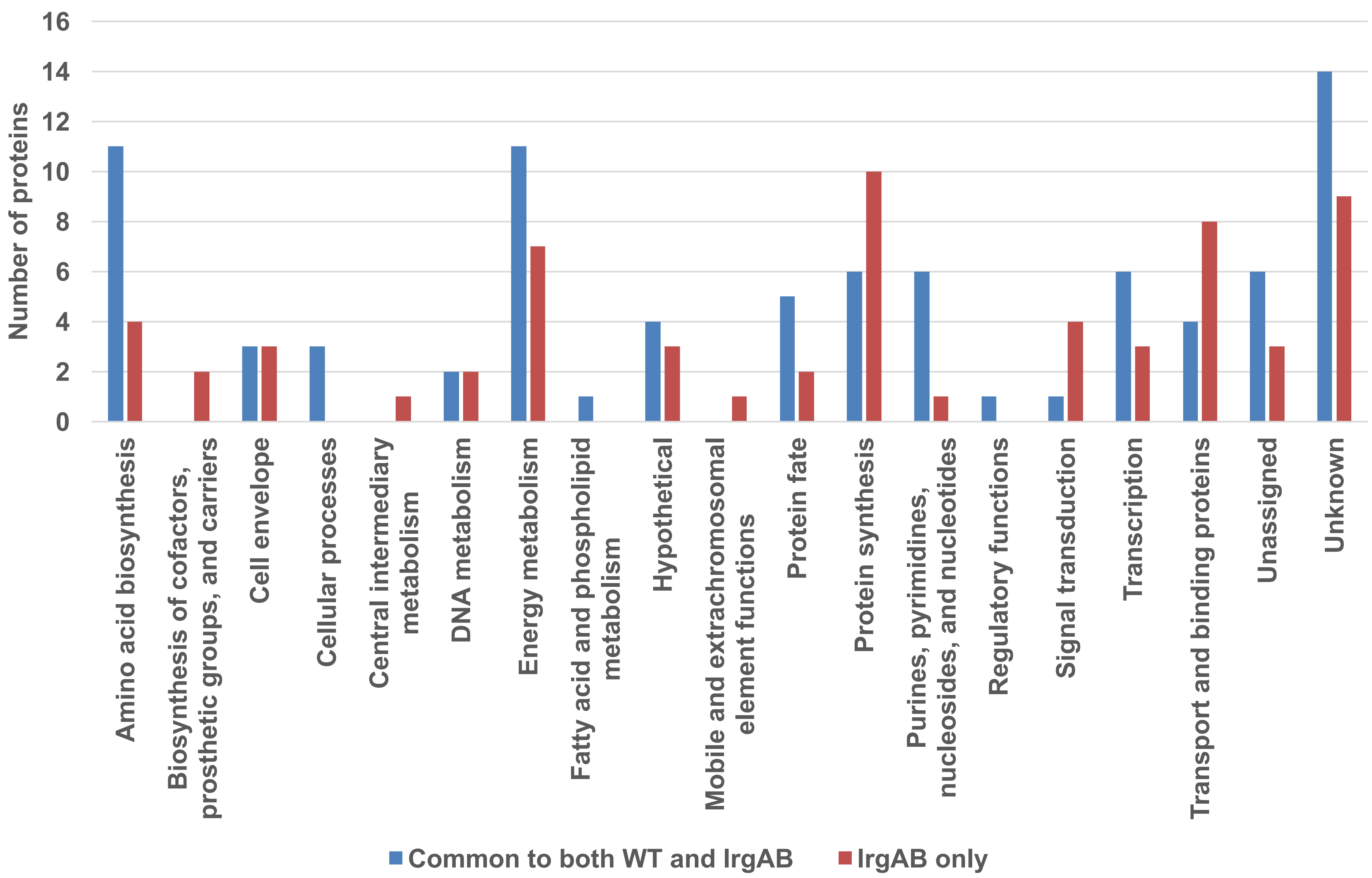

Number of proteins
16
14
12
10
8
6
4
2
0
Amino acid biosynthesis
Biosynthesis of cofactors, prosthetic groups, and carriers
Cell envelope
Cellular processes
Central intermediary metabolism
DNA metabolism
Energy metabolism
Fatty acid and phospholipid metabolism
Hypothetical
Mobile and extrachromosomal element functions
Protein fate
Protein synthesis
Purines, pyrimidines, nucleosides, and nucleotides
Regulatory functions
Signal transduction
Transcription
Transport and binding proteins
Unassigned
Unknown
Common to both WT and lrgAB
lrgAB only

Fig. S5a

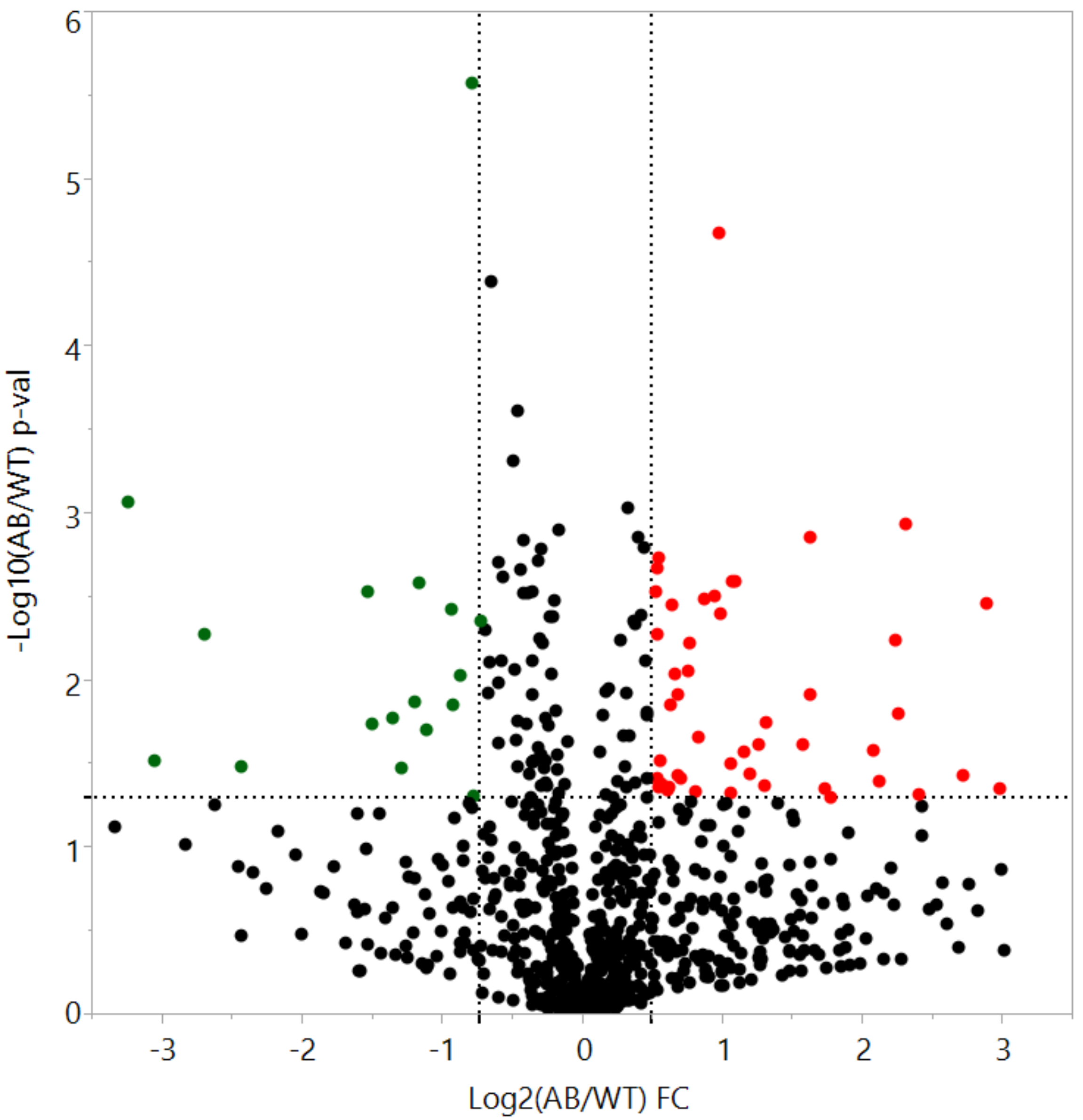

1049 proteins were identified and 76 proteins were differentially expressed with FC <0.6 or >1.4 (*p*-value<0.05)

Fig. S5b

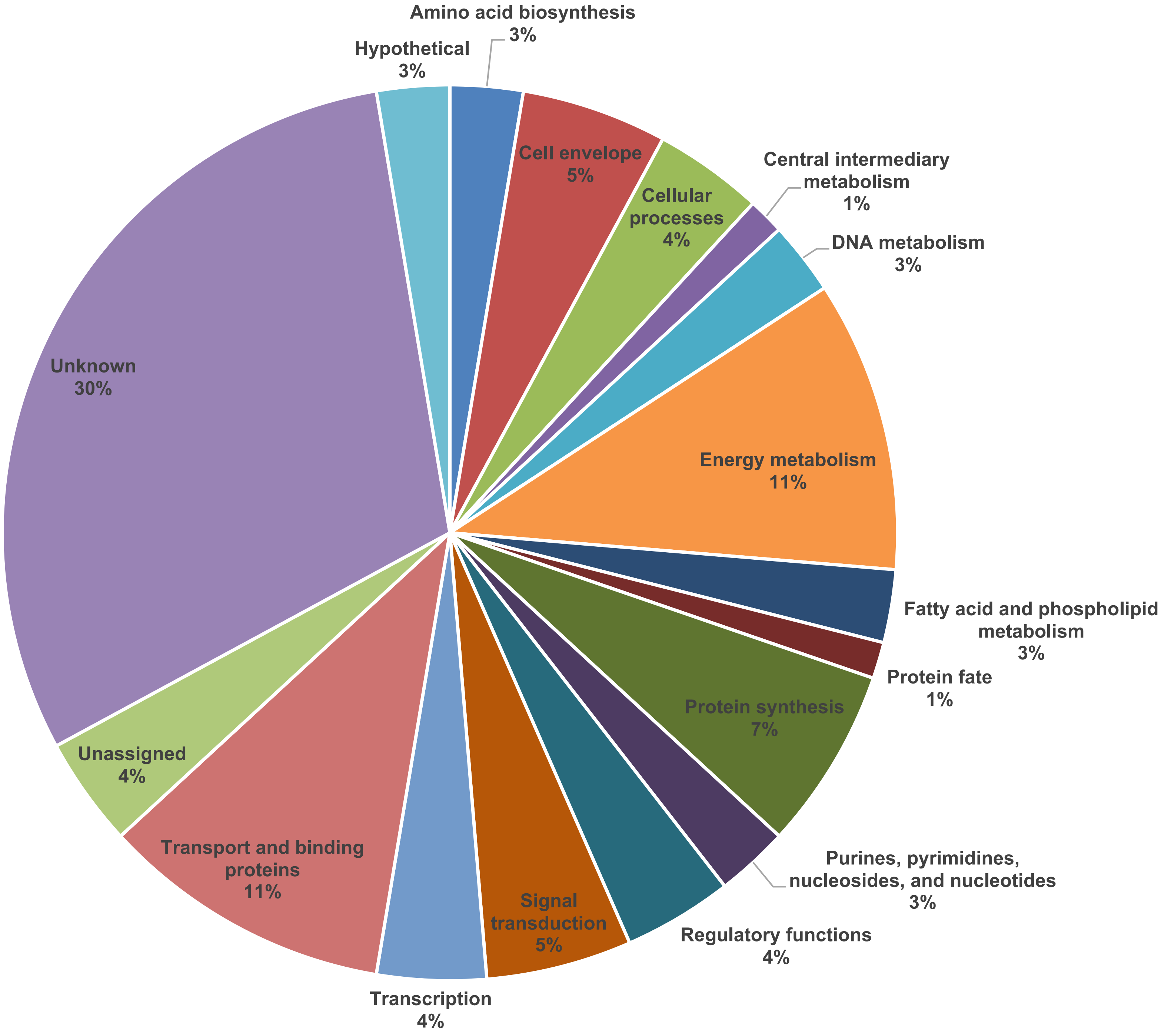
Amino acid biosynthesis
3%
Hypothetical
3%
Cell envelope
5%
Cellular processes
4%
Central intermediary metabolism
1%
DNA metabolism
3%
Energy metabolism
11%
Fatty acid and phospholipid metabolism
3%
Protein fate
1%
Protein synthesis
7%
Purines, pyrimidines, nucleosides, and nucleotides
3%
Regulatory functions
4%
Signal transduction
5%
Transcription
4%
Transport and binding proteins
11%
Unassigned
4%
Unknown
30%
